# A Near-Fatal Encounter with Acute Suicidal Behavior in Anti-N-methyl-D-aspartate Autoimmune Encephalitis

**DOI:** 10.3390/jcm13010206

**Published:** 2023-12-29

**Authors:** Eunmi Lee, Minjee Kim, Kyu-Hyouck Kyoung, Jin Yong Jun

**Affiliations:** 1 Department of Neurology, Ulsan University Hospital, University of Ulsan College of Medicine, Ulsan 44033, Republic of Korea; kimminjee627@gmail.com; 2 Department of Trauma Surgery, Trauma Center, Ulsan University Hospital, University of Ulsan College of Medicine, Ulsan 44033, Republic of Korea; traumacrew@uuh.ulsan.kr; 3 Department of Psychiatry, Ulsan University Hospital, Ulsan 44033, Republic of Korea; jjy826@uuh.ulsan.kr

**Keywords:** autoimmune encephalitis, anti-NMDAR encephalitis, epilepsy, neuroimmunology, psychosis, suicidality

## Abstract

Anti-N-methyl-D-aspartate receptor encephalitis (anti-NMDARE) is a complex neuropsychiatric syndrome known for its diverse neurological manifestations, often involving psychiatric symptoms and seizures that elevate the risk of suicidal ideation and behavior. We present a case illustrating the potentially lethal nature of anti-NMDARE, wherein an unexpected suicide attempt occurred 10 days after the onset of seizures in a 21-year-old man. Upon arrival at the emergency room, immediate interventions addressed hypovolemic shock, followed by subsequent neurosurgical and orthopedic procedures. Six days after cessation of sedation, the patient exhibited atypical focal seizures, behavioral arrest, psychotic responses, and delusions. Despite normal brain magnetic resonance imaging and cerebrospinal fluid (CSF) analysis results, a high CSF immunoglobulin G index and posterior hypometabolism on brain F-fluorodeoxyglucose positron emission tomography raised suspicion of autoimmune encephalitis. Steroids and intravenous immunoglobulins were administered. A comprehensive evaluation ruled out other conditions. Serum and CSF tests confirmed the presence of anti-NMDAR antibodies. This case highlights the potential lethality of the acute stage of anti-NMDARE, emphasizing the absence of apparent psychiatric symptoms before a suicide attempt. Further studies on suicidality associated with anti-NMDARE are crucial, underscoring the importance of vigilance in cases involving newly diagnosed seizures or psychoses.

## 1. Introduction

The diagnosis of autoimmune encephalitis (AE) is increasing because of the discovery of new neural autoantibody biomarkers and increased awareness among clinicians. Despite its overall rarity, the cumulative incidence is approximately 3–12 per million person-years. AE is increasingly considered, particularly in patients with acute psychiatric symptoms or unusual progression of new-onset epilepsy, as it is potentially treatable with immunotherapy [1,2,3]. Patients with AE often exhibit unusual seizure frequencies or atypical polymorphic psychosis, highlighting the importance of neurologists considering the probable etiology of immune disorders. Diagnosing AE can be challenging because of the wide spectrum of clinical presentations, the prevalence of psychiatric features mimicking primary psychiatric illnesses, the frequent absence of diagnostic abnormalities on conventional brain magnetic resonance imaging (MRI), non-specific findings on electroencephalography (EEG) testing, and a lack of identified immunoglobulin G (IgG) class neuronal autoantibodies in the blood or cerebrospinal fluid (CSF) in a subgroup of patients, possibly up to 50% of the all patients [4,5]. AE is usually diagnosed following the 2016 criteria proposed by Graus et al. (Table 1) [4].

Since its discovery in 2007, anti-N-methyl-d-aspartate receptor encephalitis (anti-NMDARE) has become the best-known and most common form of non-limbic AE [5,6,7,8,9]. The acute phase of this condition encompasses a polymorphic spectrum of neuropsychiatric symptoms, including behavioral problems, psychosis, delirium, catatonia, mood fluctuations, and sleep disturbances. However, limited attention has been given to the analysis of suicidal thoughts and behaviors in anti-NMDARE [10,11]. Knowledge regarding suicidal behavior and self-harm during the undiagnosed period is limited. Previous literature has described suicidality symptoms in approximately 7–13% of patients, with nearly half of these patients attempting suicide [10,12,13].

Notably, cases in which severe trauma occurs as a result of suicidal behavior pose a higher risk of remaining undiagnosed or delay the suspicion of this intrinsic neuropsychiatric syndrome. This case underscores the importance of the early involvement of neurologists alongside their psychiatric counterparts, emphasizing the challenge of a prompt and accurate diagnosis and preemptive treatment to facilitate neuropsychiatric recovery during the acute phases of new-onset epilepsy or psychosis. Herein, we report the unique case of a patient who exhibited unexpected impulsivity symptoms leading to a near-fatal suicide attempt and multiple life-threatening traumas in the context of anti-NMDA encephalitis.

## 2. Case Description

A 21-year-old man experienced three new-onset convulsive seizures within 48 h, 10 days before attempting suicide. He was initially admitted to the tertiary medical center of another region and diagnosed with new-onset focal epilepsy and initiated on antiseizure medication (levetiracetam 1000 mg/day, lamotrigine 25 mg/day). Supportive fluid therapy for rhabdomyolysis (CPK 12,603 IU/L) was administered during hospitalization, and the patient was discharged without cognitive or psychiatric symptoms a week later.

Three days after discharge, he attempted suicide by jumping from a building with a height > 10 m and was subsequently transferred to the emergency room of the trauma center. According to his parents’ report, the patient had no history of diagnosed psychiatric or psychological conflicts, neurodevelopmental problems, or substance abuse. Currently in the second year of college, he completed his mandatory two-year military service three months previously. Initial evaluations revealed deep drowsiness, inability to communicate, and unstable vital signs (blood pressure, 76/59 mmHg, pulse rate 129/min, respiratory rate 27/min) without fever, indicative of impending hypovolemic shock. The blood ethanol concentration test conducted in the emergency room yielded a negative result. Physical examination revealed scalp swelling, laceration in the right parietal area and right elbow, and coccyx abrasion. An extensive systemic evaluation revealed multiple severe trauma, including hemoperitoneum with liver laceration, omental bleeding, pneumothorax, and multiple fractures of the thoracolumbar spine, coccyx, pelvis, and right elbow. Rapid interventions, including mechanical ventilation, selective transcatheter embolization for effective hemostatic treatment, and inotropic administration, were initiated in the intensive care unit (ICU). Continuous administration of deep sedation and analgesics was employed to manage hemodynamic instability and minimize patient movement.

On the first day of hospitalization, the patient underwent an initial neurological consultation at the trauma center to establish a maintenance plan for antiseizure drug administration. Considering the infrequent severe psychiatric adverse effects associated with levetiracetam, a neurology specialist recommended the transition to a combination therapy with valproate (1800 mg/day) and a gradual titration of lamotrigine. The first EEG, performed under deep sedation, revealed no disproportionate background abnormalities, epileptiform discharges, or focal slowing. On the 6th day of hospitalization, the patient underwent extensive multi-level spinal fusion surgery for multiple thoracolumbar spinal fractures. On hospital day 7, the patient became communicative after extubation, enabling consultations and bedside patient interviews in the trauma ICU with neurologists and psychiatrists. He was oriented and reported feeling angered by his mother’s nagging, expressing that this frustration led him to jump. However, the details of this incident were inconsistent and included defensive attitudes toward the suicide attempt memory. Since the second day after regaining consciousness in the ICU, the patient exhibited intermittent eye fluttering, facial grimacing, intermittent incoherent speech, and occasional delusional episodes. EEG was repeated to exclude non-convulsive seizures. Considering the possibility of ICU delirium and post-concussional encephalopathy, olanzapine 5 mg was initiated. However, on hospital days 9–11 in the general ward, the patient’s symptoms worsened, with episodes of varied staring, unresponsiveness, eyelid fluttering, hyperventilation, and paroxysmal delusions. A second neurology consultation was requested, and following a clinical review by the neurologist, the medication regimen was recommended to be adjusted because of potential central nervous system side effects associated with the administration of high-dose narcotic analgesics for pain management. Additionally, considering the presence of postfall scalp swelling, a brain MRI was performed to exclude the possibility of focal seizures resulting from delayed cerebral edema or hemorrhage. Pain management was adjusted, and a repeat MRI ruled out diffuse axonal injury or cerebral contusions. On hospital days 12–14, no bizarre movements or critical psychotic features were observed. However, on hospital day 15, grandiose psychotic ideas and aggressive behaviors appeared, followed by emotional fluctuations. By day 20 of hospitalization, the patient was tentatively diagnosed with AE, considering the mixture of complex and atypical clinical courses of seizures and psychosis.

Extensive evaluations were conducted, encompassing various diagnostic procedures, such as brain 18F-fluorodeoxyglucose positron emission tomography (FDG-PET), tumor screening for occult malignancies, including whole-body PET-CT, testicular ultrasonography, screening for systemic autoimmune antibodies in serum, CSF analysis with assessments for viral and other infectious etiologies, detection of CSF oligoclonal bands, identification of antibodies related to central nervous system demyelination, including anti-aquaporin-4 antibodies, anti-myelin oligodendrocyte glycoprotein antibodies, paraneoplastic antibodies, and neuronal surface autoantibodies. Despite normal findings in repeated MRI and routine CSF analysis (white cell count 0/uL; protein 36 mg/dL; glucose 60 mg/dL), a high CSF IgG index (0.96) and diffuse posterior cortical hypometabolism on brain FDG-PET supported the suspicion of autoimmune etiology. From hospital day 22, preemptive high-dose steroid pulse therapy (intravenous methylprednisolone 1 g for 3 days), followed by a short tapering course of oral steroids for sequential 3 days, was administered because of multiple bone fractures, weighing the risks of potential adverse effects on bone metabolism. On hospital day 28, anti-NMDAR antibodies were confirmed (CSF and serum in cell-based assays with evidence of mouse tissue-based indirect immunofluorescence).

Subsequent intravenous immunoglobulin (0.4/kg/day for 5 days) was administered, leading to the full resolution of psychotic and cognitive symptoms in the next 2 weeks of hospitalization. A scale evaluation using the mini-mental state examination score showed 29/30, and the Montreal cognitive assessment score was 27/30. The language range of the Korean version of the Boston Naming Test and the Korean version of the Western Aphasia battery showed normal results. At the 3-month follow-up, the patient demonstrated competent recovery without physical disability. Repeat brain FDG-PET after 4 months revealed results within normal ranges, enabling the planned tapering and discontinuation of the antiepileptic medication.

**Figure 1:**
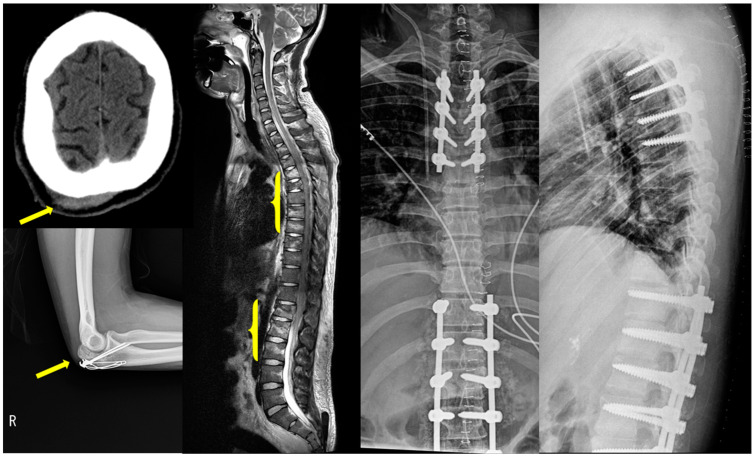
Injuries resulting from suicidal attempts. Scalp swelling and laceration, ulnar olecranon fracture and fixation, multiple thoracolumbar compression fractures, and multi-level spinal fusion are illustrated. The injury sites are highlighted in yellow.

**Figure 2:**
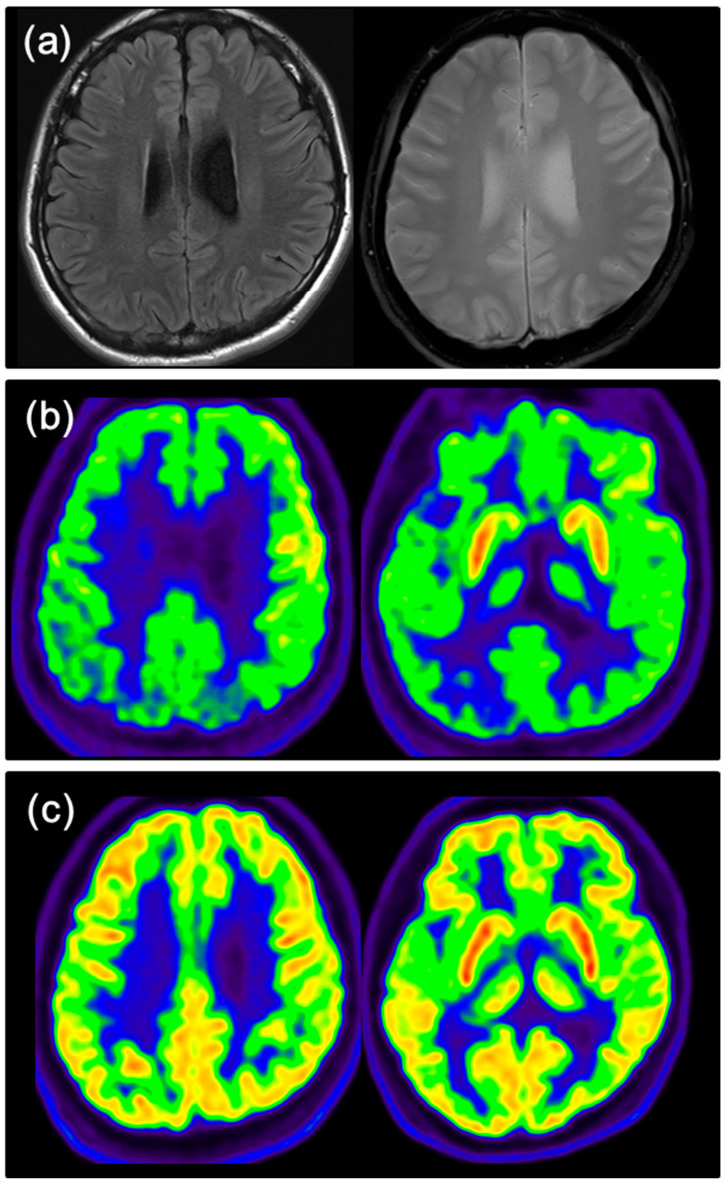
Magnetic resonance imaging depicted no abnormality in the FLAIR and GRE sequences during the acute phase of anti-NMDA receptor encephalitis (**a**). Brain 18F-fluorodeoxyglucose positron emission tomography showed a pronounced typical anteroposterior gradient pattern with marked hypometabolism in bilateral parietooccipito-temporal lobes and preserved metabolism in basal ganglia during the acute phase (**b**), which improved after a 4-month follow-up (**c**).

Multiple injuries resulting from suicide attempts are illustrated in Figure 1. The patient’s brain MRI and brain FDG-PET findings are illustrated in Figure 2.

## 3. Discussion

The patient was admitted after a near-fatal suicide attempt and multiple life-threatening traumas. During intensive trauma treatment, the medical team closely observed intricate neuropsychiatric symptoms emerging 2 weeks after the patient regained consciousness. Thorough differential diagnoses conducted by a neurology specialist ultimately led to the consideration of AE in the clinical context. The patient remarkably recovered through prompt diagnosis and immunotherapy, highlighting the importance of early recognition and intervention in anti-NMDARE cases. A definite diagnosis of anti-NMDARE was confirmed based on meeting two criteria from the presented clinical criteria, with positive serum and CSF anti-NMDAR antibodies (Table 2).

Detecting antibodies associated with AE in the CSF or serum is invaluable for aiding in the diagnostic process. However, a cautious approach is essential when interpreting antibody results to mitigate the risk of overinterpretation and subsequent misdiagnosis. This risk is particularly pronounced in cases where antibodies may not directly align with the clinical course or when the MRI or CSF findings overlap with those of differential medical diseases, potentially leading to a broad spectrum of disorders being misclassified as AE based on the possible diagnostic criteria [14]. Conversely, special attention must be directed toward cases with a high clinical suspicion of AE but lacking autoantibody positivity. Recognizing conditions such as probable seronegative AE and possible AE not otherwise categorizable, which collectively account for one-third of AE cases, holds significance in clinical practice. This recognition is crucial when considering the current limitations of laboratory tests, which are insufficient to detect all cases of AE [15].

Anti-NMDARE presents with a spectrum of symptoms, ranging from a limited form with isolated neurological or psychiatric disturbances to severe cases requiring mechanical ventilation and an extended stay in the ICU. Despite the introduction of immunotherapy and surgical resection if a tumor is identified, previous studies have reported mortality rates ranging from 5% to 11.46% [16,17]. The disease course is negatively affected by complications associated with prolonged ICU admission, and the primary causes of death include severe pneumonia, sepsis, multiple organ dysfunction syndrome, and refractory status epilepticus. Owing to the associated high morbidity and mortality, early first-line immunotherapy in patients with probable anti-NMDARE is favored without waiting for autoantibody results conformation, which might take several days [18].

This case emphasizes the potential lethality of suicidal behavior during the acute stages of anti-NMDARE. Despite initially presenting with seizures, the patient displayed no discernable psychiatric symptoms before the suicide attempt. Previous literature has focused on diverse psychiatric phenotypes, with little focus on suicidality [3,10]. Suicidality in anti-NMDARE relates to mortality from a different perspective than complications associated with delayed detection or a poor immunotherapy response. Existing studies focusing on suicidality-associated anti-NMDARE predominantly reported a high prevalence of suicidality occurring before admission, with patients initially presenting with more prominent psychiatric symptoms [12,13]. However, in this particular case, the family consistently accompanied the patient from the 1-week hospitalization for new-onset seizures until the suicide attempt. If psychiatric symptoms or emotional changes had been present, the family would have noticed them. Therefore, this scenario, in which the initial psychiatric manifestation involves suicide attempts without preceding prodromal psychosis or mood changes, is unusual. If the patient’s suicide attempt had resulted in death or severe traumatic brain injury, the underlying AE might have remained undetected, leading to psychological shock and emotional distress for the family. As suicidal thoughts and behaviors represent a potentially lethal risk, clinicians initially evaluating patients with newly diagnosed epilepsy or the first episode of psychosis or those suspected of having anti-NMDARE must rigorously monitor suicide risk during the acute or unrevealed phase of the disease.

In this case, ongoing correction of clinical reasoning for an accurate diagnosis is a crucial aspect. A broad range of differential diagnoses must be explored and ruled out during the diagnostic workup, as many disorders can mimic AE. Therefore, achieving a diagnosis requires a high index of clinical suspicion after reasonably excluding alternative causes (Table 3).

The presence of multiple severe traumas resulting from suicidal behavior adds complexity to the differential diagnosis of patients with possible AE. The most relevant alternative disorder to consider in this patient evolved over time. Initially, a perceived bias toward the diagnosis of epilepsy based on an event 10 days prior at another hospital led to the prioritization of postictal psychosis or psychiatric side effects of antiseizure drugs as potential triggers for suicide attempts. Levetiracetam has been reported to cause varying degrees of adverse psychiatric effects [5]. After the multi-level spinal fusion surgery, the patient’s neuropsychiatric symptoms became evident on the seventh day of hospitalization. Considering the prolonged sedation period, we sequentially considered the possibility of the coexistence of post-concussional amnesia, ICU delirium, and post-traumatic stress disorder. Nevertheless, persistent neuropsychiatric symptoms led to the consideration and exclusion of the possibility of CNS side effects from high-dose narcotics, post-traumatic axonal injury, or cerebral contusion. On the 15th day of hospitalization, as the patient continued to exhibit a complex array of symptoms, including behavioral changes, mood fluctuations, and psychosis, the clinical reasoning ultimately shifted toward the possibility of AE. The step-by-step exclusion of diagnoses demonstrates the importance of comprehensive reassessment of unknown causes and timely decisions.

In addition to clinical reasoning, FDG-PET played a crucial role in supporting the clinical diagnosis in this case. Despite the absence of distinct cerebral atrophy or encephalitis-suggestive lesions on neuroimaging, a significant decrease in brain metabolism in the bilateral parietotemporo-occipital lobes, inconsistent with the patient’s age, raised suspicions of brain structure-functional dissociation. This, coupled with the absence of clear infectious or inflammatory markers in the CSF analysis results, strongly suggested an immune etiology. These FDG-PET findings were pivotal in establishing clinical confidence in AE and in initiating an active immunotherapeutic strategy before confirmation through autoantibody testing. Conventional MRI lacks sensitivity for diagnosing anti-NMDARE, as brain structural abnormalities are often absent on initial presentation or follow-up MRI of the brain in up to 80% of individuals with anti-NMDARE, even in those with profound neurological deficits [18,19,20]. In contrast, FDG-PET abnormalities are common but etiologically non-specific to brain metabolic abnormalities, including those observed in degenerative CNS diseases or encephalitis. The typical hypometabolism of associative posterior areas, mainly the occipital region, was described as an early biomarker for distinguishing anti-NMDARE from other AE [21]. FDG-PET hypermetabolism may indicate an active and persistent neuroinflammatory process in the proper clinical context or the ictal phases related to encephalitis. In most cases, a mixed pattern of basal ganglia hypermetabolism and diffuse cortical hypometabolism depends on the phase of the disease [22]. When brain MR findings are absent but clinical findings suggest the possibility of AE, brain FDG-PET imaging may be warranted, particularly early in the disease process when clinical suspicion of AE is high [19].

## 4. Conclusions

Suicidality is one of the most fatal complications associated with anti-NMDARE. Physicians should maintain a heightened awareness of the grave risk of suicidality due to newly developed seizures, psychotic symptoms, and deteriorating neuropsychiatric conditions that may indicate AE onset. Further studies and increased interest are warranted to explore suicidality in anti-NMDARE. Collaborative, timely diagnostic and differential decision-making processes, coupled with meticulous attention from a multidisciplinary approach during critical stages, are crucial for achieving a rapid diagnosis and facilitating neuropsychiatric recovery.

## Figures and Tables

**Table 1:** Diagnostic criteria for possible autoimmune encephalitis [4].

Diagnosis can be made when all three of the following criteria have been met: 1. Subacute onset (rapid progression of less than 3 months) of working memory deficits (short-term memory loss), altered mental status *, or psychiatric symptoms
2. At least one of the following: New focal CNS findings; Seizures not explained by a previously known seizure disorder; CSF pleocytosis (white blood cell count of more than five cells per mm^3^); MRI features suggestive of encephalitis ^†^.
3. Reasonable exclusion of alternative causes

* Altered mental status is defined as a decreased or altered level of consciousness, lethargy, or personality change. ^†^ Brain MRI hyperintense signals on T2-weighted fluid-attenuated inversion recovery sequences highly restricted to one or both medial temporal lobes (limbic encephalitis) or in multifocal areas involving grey matter, white matter, or both compatible with demyelination or inflammation. Abbreviations: CNS, central nervous system; CSF, cerebrospinal fluid; MRI, magnetic resonance imaging.

**Table 2:** Diagnostic criteria for anti-NMDA receptor encephalitis [4].

Probable anti-NMDA receptor encephalitis * Diagnosis can be made when all three of the following criteria have been met:	
Criterion	Findings in this patient
1. Rapid onset (<3 months) of ≥4 of the 6 following major groups of symptoms:	
Abnormal (psychiatric) behavior or cognitive dysfunction	No apparent psychiatric symptom before hospitalization. Delusion and aggressive behavior during hospitalization
Speech dysfunction (pressured speech, verbal reduction, mutism)	None
Seizures	Known new-onset seizure disorder; convulsive seizures 10 days before suicidal attempt followed by new strange focal seizure during hospitalization
Movement disorder, dyskinesias, or rigidity/abnormal postures	None
Decreased level of consciousness	None
Autonomic dysfunction or central hypoventilation	None
2. Presence of ≥1 of the following laboratory study results:	
Abnormal EEG (focal or diffuse slow or disorganized activity, epileptic activity, or extreme delta brush)	Sedative background on hospital day 5 Normal background with intermittent diffuse mild slowing on hospital day 7 but non-specific finding or confounded by narcotics usage, severe multiple trauma, and ICU care state
CSF with pleocytosis or oligoclonal bands	None Negative finding for oligoclonal band
	Cryptogenic epilepsy [not immune-related] with postictal psychosis Psychiatric side effects of levetiracetam Post-concussional amnesia ICU delirium Post-traumatic stress disorder Diffuse axonal injury and cerebral contusion CNS side effects of high-dose narcotics
3. Reasonable exclusion of other disorders	
Diagnosis can also be made in the presence of three of the above groups of symptoms accompanied by a systemic teratoma.	Not applicable to men
Definite anti-NMDA receptor encephalitis *	
Diagnosis can be made in the presence of one or more of the six major groups of symptoms and IgG anti-NMDAR1 (GluN1) antibodies, ^†^ after reasonable exclusion of other disorders.	

* Patients with a history of herpes simplex virus encephalitis in the previous weeks might have relapsing immune-mediated neurological symptoms (post-herpes simplex virus encephalitis). ^†^ Antibody testing should include testing of CSF. If only serum is available, confirmatory tests should be included (for example, live neurons or tissue immunohistochemistry should be included in addition to cell-based assay). Abbreviations: EEG, electroencephalography; CNS, central nervous system; CSF, cerebrospinal fluid; ICU, intensive care unit; NMDA, N-methyl-D-aspartate.

**Table 3:** Differential diagnosis in patients with possible autoimmune encephalitis [4].

Central nervous system infections	Septic encephalopathy
Metabolic encephalopathy	Drug toxicity *
Cerebrovascular disease	Neoplastic disorders
Creutzfeldt–Jakob disease	Epileptic disorders
Rheumatologic disorders (e.g., lupus, sarcoidosis, other)	Kleine–Levin
Reye syndrome (children)	Mitochondrial diseases
Inborn errors of metabolism (children)	

* Includes use of illicit drugs, the direct neurotoxic effect of prescribed drugs or through induction of seizures, posterior reversible encephalopathy, idiosyncratic reaction (e.g., neuroleptic malignant syndrome), drug interaction (e.g., serotoninergic syndrome), or drug withdrawal.

## Data Availability

No new data were created or analyzed in this study. Data sharing is not applicable to this article. The data are not publicly available due to privacy considerations.
